# Nuclear RNA-binding proteins meet cytoplasmic viruses

**DOI:** 10.1261/rna.080313.124

**Published:** 2025-03

**Authors:** Alfredo Castello, Wael Kamel

**Affiliations:** MRC-University of Glasgow Centre for Virus Research, Glasgow G61 1QH, United Kingdom

**Keywords:** antiviral, RNA, RNA-binding protein, U2 snRNP, virus

## Abstract

Cytoplasmic viruses interact intricately with the nuclear pore complex and nuclear import/export machineries, affecting nuclear-cytoplasmic trafficking. This can lead to the selective accumulation of nuclear RNA-binding proteins (RBPs) in the cytoplasm. Pioneering research has shown that relocated RBPs serve as an intrinsic defense mechanism against viruses, which involves RNA export, splicing, and nucleolar factors. For instance, the U2 small nuclear ribonucleoprotein (snRNP) relocates to the cytoplasm in infected cells and uses U2 snRNA to interact with viral genomes, repressing viral replication and gene expression. Here, we describe these emerging host–virus interactions and discuss the remaining questions to elucidate their antiviral mechanisms.

## AN OVERVIEW OF RNA METABOLISM IN VIRUS-INFECTED CELLS

RNA is a pivotal molecule for RNA viruses, as it functions not only as messenger (m)RNA but also as a genome. It is thus unsurprising that viral RNA is a critical hub for host–virus interactions (Iselin et al. 2022; Castello et al. 2024). Because the limited coding capacity of viral genomes, viruses rely on host factors to replicate and spread. Most RNA viruses replicate in the cell's cytoplasm, with important exceptions such as retroviruses as the human immunodeficiency virus (HIV), or influenza virus (IV) among others (Te Velthuis and Fodor 2016; Muller et al. 2022). After entry, the viral capsid disassembles and releases the viral genome that in positive stranded viruses is translated to produce the RNA-dependent RNA polymerase (RdRp) complex. The RdRp then replicates the genomic RNA by first producing an antisense negative strand that is used as a template to generate more copies of the positive strand (i.e., genome). In some instances, viruses use a plethora of mechanisms to generate shorter positive stranded RNAs encoding viral proteins that must be expressed with different stoichiometry or/and timing than the main open reading frame (Ou et al. 1982; Kim et al. 2020; Truman et al. 2020). Negative stranded viruses, however, cannot translate their genomes as these are antisense, and thus the viral particle carries the replication machinery from the cell where the virus was packaged. Replication of cytoplasmic viruses typically occurs in membranous compartments that can be derived from different subcellular organelles, particularly the endoplasmic reticulum (ER) and endosomes (Nagy et al. 2016; Romero-Brey and Bartenschlager 2016). Conversely, a smaller group of viruses employ membraneless granules that are formed by protein condensates, as is the case for the respiratory syncytial virus (RSV) (Sagan and Weber 2023). Either way, viral replication organelles (VORs) are typically physically separated from the intracellular milieu limiting the availability of viral RNA for antiviral sensing.

Viruses interplay with virtually all the steps of the life cycle of cellular RNAs that begins with their genesis mediated by cellular RNA polymerases. Transcription represents a threat for viruses, as the activation of viral RNA sensors causes the transcriptional activation of the interferon genes (Barbalat et al. 2011; Jensen and Thomsen 2012). Therefore, it is not surprising that viral proteins can interfere with cellular transcription to prevent interferon signaling (Garmashova et al. 2006; Rajani et al. 2012; Banerjee et al. 2020). Other viruses, however, must keep cellular transcription active as they acquire the cap structure of viral RNA from cellular mRNAs in a process known as cap snatching (De Vlugt et al. 2018; Xu et al. 2022). Retroviruses, including the HIV, are also reliant in cellular transcription as their RNAs are produced by the cellular RNA polymerase II.

Cytoplasmic RNA viruses do not rely on the RNA splicing machinery. However, nuclear RNA viruses such as IV and HIV rely on the splicing of their RNAs to produce the full repertoire of viral transcripts and, consequently, proteins (Dubois et al. 2014; Truman et al. 2020). In contrast, several splicing factors were reported to translocate to the cytosol in cells infected with cytoplasmic viruses (Tapescu et al. 2023; Zheng et al. 2023; Kamel et al. 2024), and the significance of this phenomenon will be expanded in greater detail below.

Nuclear-cytoplasmic trafficking is also key for both nuclear and cytoplasmic viruses. Several mechanisms employing viral proteins to disrupt the nucleo/cytoplasmic trafficking were described for cytoplasmic viruses. For example, the protein M of vesicular stomatitis virus (VSV) and proteases of enteroviruses target nucleoporins (Nup), particularly Nup98 (von Kobbe et al. 2000; Gustin and Sarnow 2002; Castello et al. 2009; Watters and Palmenberg 2011; Watters et al. 2017). This is an example of convergent evolution, where two highly diverse viruses use similar strategies to impair nuclear-cytoplasmic communications. In both cases, disruption of nucleo/cytoplasmic trafficking impedes the import of antiviral transcription factors and prevents nuclear export of newly transcribed mRNAs encoding the antiviral program (von Kobbe et al. 2000; Castello et al. 2009). Whether the manipulation of the nuclear pore complex (NPC) happens with other cytoplasmic viruses remains poorly understood. Nuclear viruses, however, must maintain an operative NPC and trafficking machinery to enable the export of viral ribonucleoproteins (RNPs) to the cytoplasm. Viral RNA export may involve alternative pathways coopted by viral proteins. For example, HIV Rev hijacks the export factor CRM1 (also known as Exportin 1 [XPO1]), which is critical to translocate ribosomal subunits and other cargos out of the nucleus (Kohler and Hurt 2007; Truman et al. 2020).

Protein synthesis represents a key step of host-dependency because of the magnitude of functions present in the translation apparatus that cannot be encoded into small viral genomes (Castello et al. 2024). Most cytopathic viruses ensure efficient viral protein synthesis by inducing host protein synthesis shutoff (Carrasco et al. 2018). How mRNA translation is suppressed depends upon the virus, for example poliovirus cleaves the eukaryotic initiation factor (eIF)4G that acts as a molecular bridge recruiting the translation machinery to the mRNA in a cap-dependent manner (Gradi et al. 1998; Prevot et al. 2003), while Sindbis virus (SINV) induces phosphorylation of eIF2α that hampers the delivery of the initiator methionine tRNA to the ribosome (Gradi et al. 1998; Ventoso et al. 2006). Viral RNAs bypass protein synthesis shutoff through the use of unorthodox translation mechanisms, including internal ribosome entry sites (Prevot et al. 2003; Martinez-Salas and Fernandez-Miragall 2004; Gebauer and Hentze 2016) and noncanonical translation initiation complexes (Walsh and Mohr 2006; Toribio et al. 2016; Carrasco et al. 2018).

RNA sequestration and degradation also interplay with viral RNAs. Stress granules can sequester viral RNA into a translation and replication suppressed state (Onomoto et al. 2014; Jayabalan et al. 2023). However, some viruses have developed mechanisms to subvert this control and, in some instances, repurpose stress granule proteins. For example, SINV nsP3 sequesters G3BP proteins prevent the assembly of stress granules and the sequestration of viral RNA (Lu et al. 2021; Brownsword and Locker 2023; Jayabalan et al. 2023). Viruses also interplay with other membraneless organelles such as p-bodies (Mok et al. 2012; Lloyd 2013) and the nucleolus (Kamel et al. 2024; Ulloa-Aguilar et al. 2024), although the role of these structures in infection and their potential for viral RNA sequestration remain under intensive debate. The p-body associated protein XRN1 is a 5′-3′ exonuclease that is key in cellular RNA decay and has been involved in virus infection, with proposed virus-dependency and antiviral roles. The antiviral role of XRN1 was associated with its ability to degrade viral RNA, particularly upon activation of the antiviral endonuclease RNaseL (Odon et al. 2019). However, XRN1 was also linked to the removal of double-stranded (ds)RNA, preventing detection of viral RNA by antiviral sensors (Liu et al. 2021; BenDavid et al. 2022). In flavivirus infection, XRN1 degrades viral RNA but it is stalled onto a pseudoknot in the 3′ UTR of the viral genome, giving rise to a degradation-dependent subgenomic RNA with the capacity to antagonize antiviral proteins (Liu et al. 2023). Moreover, the cellular transcriptome is pervasively remodeled upon the infection by other pathogenic viruses, such as SINV and the causal agent of COVID-19, SARS-CoV-2 (Garcia-Moreno et al. 2019; Shehata and Parker 2023). It is believed that XRN1 is critical for the decay of cellular RNAs in virus-infected cells, which can potentially contribute to the translation shutoff. A recent work suggested that degradation of cellular mRNAs can increase the available pool of free nucleotides to promote rapid viral replication (Ruscica et al. 2024).

Cellular RNA-binding proteins (RBPs) are also central to the cell's antiviral arsenal. The cell harbors a set of highly specialized RBPs that selectively recognize signatures present in viral RNAs called pathogen-associated molecular patterns (PAMPs). PAMPs include unusual molecular signatures such as double-stranded (ds)RNA, 5′ triphosphate ends, unmethylated caps, and sequence biases (Taylor et al. 2005; Vladimer et al. 2014; Li et al. 2017; Shaw et al. 2021). Antiviral sensor activation triggers the transcription of interferons or, alternatively, can exert an antiviral function directly on viral RNAs to suppress infection (Gack et al. 2007; Barbalat et al. 2011; Jensen and Thomsen 2012; Vladimer et al. 2014).

Here, we discuss an exciting and poorly understood phenomenon involving a selective group of nuclear RBPs that migrate to the cytoplasm upon infection with cytoplasmic viruses and repress viral replication and gene expression (Lloyd 2015; Tapescu et al. 2023; Zheng et al. 2023; Kamel et al. 2024).

## A SUBSET OF NUCLEAR RBPs TRANSLOCATE TO THE CYTOPLASM OF VIRUS-INFECTED CELLS

**FIGURE 1.**
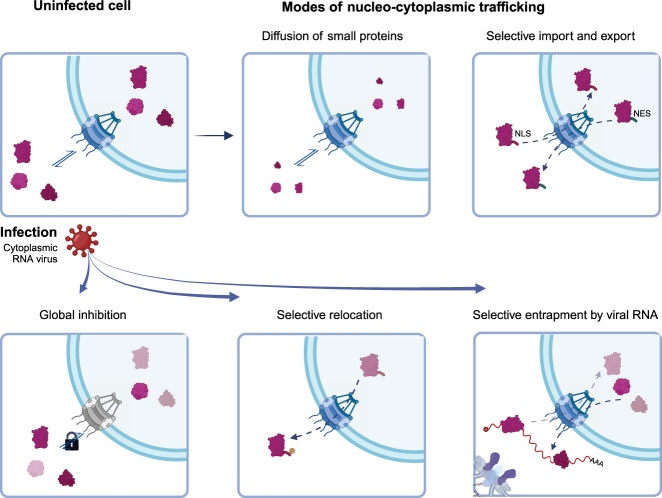
Mechanisms that can contribute to the regulation of nucleocytoplasmic trafficking of proteins in virus-infected cells. Schematic representation of global and specific mechanisms controlling protein localization (*top* panels). Viruses can affect the trafficking between the nucleus and the cytoplasm globally by targeting key proteins regulating the NPC, as is the case of Nup98 that is inhibited by enteroviral 2A proteases and VSV M (*bottom left* panel). Selective relocation often involves a loss or gain of posttranslation modifications in NLSs or NESs, altering the function of these motifs and promoting a nuclear or cytoplasmic localization of specific proteins (*bottom central* panel). Alternatively, viral RNA can function at spiderweb that sequesters shuttling proteins at the VROs (*bottom right* panel). Viral RNA is very abundant in infected cells, and its high local concentration could promote binding of RBPs, even in low-affinity ranges.

The cell's cytoplasm and nucleus are separated by the nuclear envelope, and trafficking between these two subcellular compartments is mediated by the NPC. While small molecules (including proteins) can traverse between the two compartments by diffusion through the NPC, large molecules and macromolecular complexes require the activity of cellular adaptors, importins, and exportins to mediate selective engagement with the NPC for nuclear import and export, respectively (Moroianu 1999; Kohler and Hurt 2007). Protein engagement with the trafficking machinery is mediated by nuclear localization signals (NLS) or nuclear export signals (NES) in the cargo protein (or complex), and interplay between NESs and NLSs can lead to complex shuttling dynamics between nucleus and cytoplasm (Moroianu 1999). Early studies showed an intricate connection between viruses and the NPC, including viral proteins, such as the enteroviral 2A protease and VSV M, that target nucleoporins and export/import adaptors (von Kobbe et al. 2000; Gustin and Sarnow 2002; Castello et al. 2009; Watters and Palmenberg 2011; Lloyd 2015; Watters et al. 2017). These mechanisms reflect the strategic importance for cytoplasmic viruses to hijack the NPCs and, consequently, the communication channels between the nucleus and the cytoplasm. By hampering the NPC and trafficking machinery, cytoplasmic viruses prevent key transcription factors in the antiviral program to be imported to the nucleus and newly synthesized RNAs encoding antiviral proteins to be exported to the cytoplasm (Fig. 1A; von Kobbe et al. 2000; Gustin and Sarnow 2002; Castello et al. 2009; Watters and Palmenberg 2011; Watters et al. 2017). The cytokine interferon-gamma (IFN-γ) induces the overexpression of two key proteins that are commonly targeted by viruses, Nup98 and Rae1, which relieves the nucleo/cytoplasmic trafficking block imposed by enteroviruses and VSV (Enninga et al. 2002; Faria et al. 2005; Castello et al. 2009). The arms race between host cells and viruses to control nuclear-cytoplasmic communication has been explored in only a few experimental models, deserving further attention.

Initial work in enteroviruses and VSV focused on their global effects in NPC dysregulation, which affects most proteins and RNAs (von Kobbe et al. 2000; Gustin and Sarnow 2002; Castello et al. 2009; Watters and Palmenberg 2011; Watters et al. 2017). However, more specific effects were observed with other viruses such as SINV, which triggers the accumulation of TIA1 and PTBP in the cytoplasm of infected cells (Sanz et al. 2015). There are two possible explanations for the accumulation of a limited set of proteins in the cytoplasm: (i) a partial inhibition of NPC function that affects predominantly proteins with high shuttling activity, or (ii) a selective translocation of specific proteins to the cytoplasm through regulation of NLS/NES, potentially induced by the stress signaling associated with virus infection (Fig. 1). In the first scenario, nuclear RBPs are present in the cytoplasm as a by-product of the abrogation of nucleo/cytoplasmic trafficking, while in the second scenario the translocation is specific of a group of proteins. Several studies pointed to a potential role of delocalized proteins in infection. For example, TIA1 and PTBP accumulation in the cytoplasm of SINV-infected cells has been linked to SINV-induced shutoff of protein synthesis (Carrasco et al. 2018). eIF2α is phosphorylated by the protein kinase R (PKR) upon SINV infection, which was considered as the main cause of protein synthesis shutoff (Ventoso et al. 2006). Surprisingly, inhibition of host protein synthesis also occurs in PKR-deficient cells in conditions where eIF2α is not phosphorylated (Sanz et al. 2015). An interesting observation was that cytoplasmic accumulation of nuclear RBPs occurred in all conditions under which shutoff was observed including in PKR null cells (Sanz et al. 2015). This led to the speculation that accumulation of nuclear proteins in the cytoplasm could interfere with cytoplasmic steps of the RNA life, although the exact mechanisms underpinning this phenomenon remain unknown.

The specificity of cytoplasmic accumulation of nuclear RBPs in SINV-infected cells was recently tackled systematically in a study that used a multi-proteomic approach to elucidate the composition of viral RNPs (Kamel et al. 2024). Viral RNA interactome capture (vRIC) was used to identify the proteins that interact with SINV RNAs in infected cells, revealing hundreds of cellular RBPs. This analysis was combined with nucleo/cytoplasmic fractionation and quantitative mass spectrometry to profile the subcellular location of RBPs in infected cells. The analysis revealed that most nuclear proteins remain nuclear upon infection, ruling out the idea of nuclear leakiness or global effects on protein localization due to NPC shutdown. Conversely, a selective group of cellular proteins accumulated in the cytoplasm, most having in common that they interact with SINV RNAs (Kamel et al. 2024). Microscopy experiments revealed a striking colocalization of several of these nuclear RBPs with VROs in the cytoplasm of infected cells (Garcia-Moreno et al. 2019; Kamel et al. 2024). Altogether, the presence of the relocated RBPs in the VROs and their interaction with the viral RNA further confirmed that these proteins do not sit idle in the cytoplasm but engage with viral processes. These RBPs belong to three main groups: nucleolar components (e.g., NOP2, NOP16, NOP53), splicing factors (e.g., SF3B1, U2AF2, SSRM1, AQR, MATR3), and RNA export machinery (e.g., DDX39A, NCBP1) (Tapescu et al. 2023; Kamel et al. 2024). This first systematic analysis revealed a wider but still highly specific set of nuclear RBPs in the cytoplasm of infected cells, most of which have no known links with virus infection. Whether specific protein translocation to the cytoplasm is a by-product of a partial shutdown of the NPC or to specific signaling controlling NLSs and NESs requires further attention.

Viral RNA is very abundant in SINV VROs, while cellular mRNAs are degraded as a consequence of the shutoff of protein synthesis (Garcia-Moreno et al. 2019). An alternative (but not mutually exclusive) hypothesis is that the high concentration of viral RNA in VROs contributes to the retention of shuttling RBPs in the cytoplasm, acting as molecular “spiderwebs” (Fig. 1). The high local concentration of viral RNA in VROs could cause a shift toward the RNA-bound state of these proteins even if their interactions are of low affinity (Jankowsky and Harris 2015). This hypothesis is compatible with the outstanding colocalization of several of the relocated nuclear RBPs with viral RNA in VROs (Garcia-Moreno et al. 2019; Kamel et al. 2024). High local concentration of RNA could potentially tether the nuclear RBPs to the membrane-bound compartments where viruses replicate their genomes and translate their mRNAs. However, the importance of viral RNA in nuclear RBP retention remains untested.

## A HIDDEN ANTIVIRAL NETWORK COMPOSED BY NUCLEAR RBPs?

In a naïve cell infected by a virus, antiviral sensors are activated upon their interaction with viral PAMPs, which then triggers interferon synthesis. Interferon molecules interact with the interferon receptors initiating a signaling cascade that ends in the synthesis of interferon-stimulated genes (ISGs) (Schoggins et al. 2011; Yang and Li 2020). ISGs include not only sensors, but also effector molecules that target a wide range of processes in the viral life cycle to suppress infection. Many viruses have developed sophisticated mechanisms to prevent the activation of antiviral sensors. They achieve this by either reducing the number of PAMPs available for detection by “hiding” viral genomes and replication intermediates in membrane-bound compartments, or by encoding viral proteins that antagonize antiviral factors (Nagy et al. 2016; Romero-Brey and Bartenschlager 2016; Chen et al. 2024). Therefore, infection results in a race between the host cell aiming at detecting the viral RNA, and the virus rushing to create the firewalls that could prevent its detection. Eventually, a cytopathic virus will cause a global shutoff of cellular gene expression, and the cell may no longer be able to respond to the infection. Therefore, having intrinsic cellular mechanisms to delay infection can provide the required time for the antiviral defenses to sense the virus and, consequently, induce the synthesis of interferon. Growing evidence suggests that relocated nuclear RBPs may represent an intrinsic defense line against invading viruses.

Interestingly, many of the proteins that translocate to the cytoplasm in SINV-infected cells substantially reduce virus infection, although whether they function in a coordinated multilayered antiviral network or individually was not explored in detail (Tapescu et al. 2023; Kamel et al. 2024). DDX39A was recently reported to translocate to the cytoplasm also in Chikungunya virus (CHKV) infection, an alphavirus related to SINV (Tapescu et al. 2023). The translocation of DDX39A caused a potent inhibition of CHKV replication, which was not reverted by ruxolitinib treatment. This drug is a potent antagonist of the interferon pathway that inhibits the Janus kinase (JAK), suggesting that DDX39A antiviral activity is independent of interferon signaling (Tapescu et al. 2023). The antiviral effects of DDX39A were observed already at 4 h post infection, which is compatible with an intrinsic antiviral mechanism with the objective of delaying virus infection to facilitate viral sensing and interferon induction. DDX39A partially relocates to the cytoplasm of CHKV-infected cells although colocalization with replication centers is only sparse. It interacts with the conserved structure element (CSE) at the 5′ region of the viral genome and stem–loop 3 (SL3), both important for replication (Tapescu et al. 2023). Interestingly, mutations in the CSE reduced virus sensitivity to DDX39A suggesting that its interaction with this sequence is critical for viral suppression.

**FIGURE 2.**
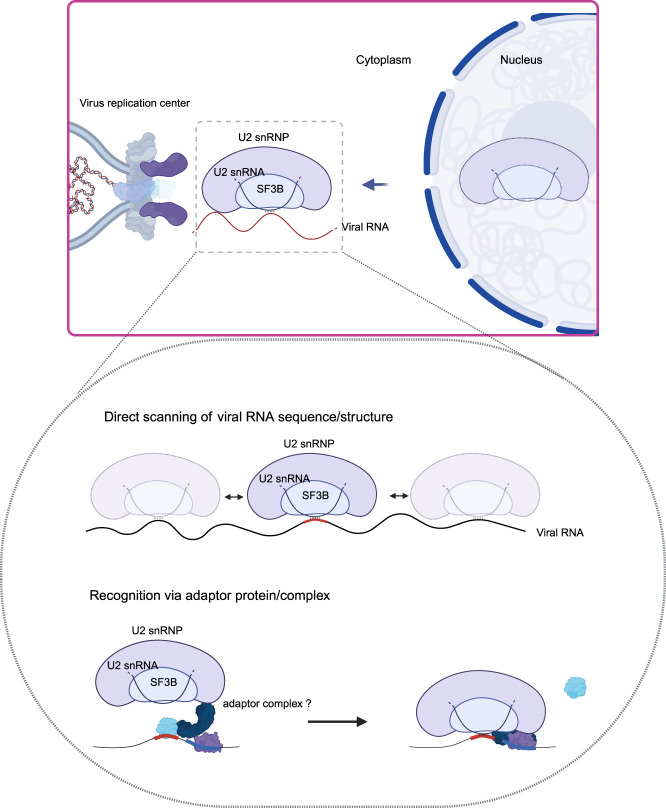
The U2 snRNP moonlights as an antiviral complex. Schematic representation of the working model by which the U2 snRNP might suppress the infection of cytoplasmic viruses. It represents two potential working models on how the relocated U2 snRNP components recognize viral RNA: (1) through a direct scanning of viral RNA seeking for accessible complementary sequences to the U2 snRNA, and (2) recruitment of the U2 snRNP to the targeted regions by adaptor complexes with high specificity. For example, U2AF1, U2AF2, and SF1 play a crucial role in branching point recognition, which enables the recruitment of the U2 snRNP (Wahl et al. 2009). A similar mechanism may occur at VROs, with U2AF1–U2AF2–SF1 or an unknown complex depositing the U2 snRNP at a branching point-like sequence on the viral RNA.

The U2 small nuclear ribonucleoprotein (U2 snRNP) also possess a moonlighting antiviral function. U2 snRNP is central for pre-mRNA splicing, being essential for the formation of the lariat at the branching point of the intron (Sun 2020). U2 snRNP includes the SF3B complex, U2 snRNA, and other protein cofactors. The majority of U2 snRNP components accumulate in the cytoplasm of SINV-infected cells (Fig. 2). However, U2 snRNP does not concentrate in the cytosol but in VROs, and complementary approaches showed that it interacts with viral RNA and proteins (Kamel et al. 2024). Interestingly, perturbation of U2 snRNP activity by knocking down its core component SF3B1 or using a specific inhibitor called Pladienolide B, massively enhances the infection of several alphaviruses, including SINV, and the picornavirus coxsackievirus B3 (Kamel et al. 2024). Conversely, no stimulation of virus infection was observed when downstream steps in the splicing reaction were inhibited, supporting that the antiviral activity of the U2 snRNP is independent of the splicing of cellular mRNAs (Kamel et al. 2024). Importantly, the stimulation of virus infection was recapitulated by interfering with U2 snRNA binding to target sequences with antisense oligonucleotides. This suggested an RNA-mediated antiviral mechanism involving the interaction of the U2 snRNA plausibly with SINV RNA (Fig. 2). Such interaction was confirmed by psoralen crosslinking and U2 snRNA purification, implying base-pairing with the viral RNA. These results explain the presence of phosphorylated SF3B1 at the VROs, which requires the interaction of U2 snRNP with its RNA substrate (Sun 2020; Kamel et al. 2024). Binding of snRNAs to viral genomes has also been reported by global studies examining RNA–RNA interactions in SARS-CoV-2 and Zika virus (ZIKV) infected cells (Ziv et al. 2018; Zhao et al. 2024). An open question was how U2 snRNP recognizes the viral RNA specifically. The study observed that viral genomes have in average more branching point-like sequences (∼11 per alphavirus or picornavirus genome) than introns (∼6) and exons (∼0.3) (Kamel et al. 2024). A potential explanation for the high incidence of branching point-like sequences is that cytoplasmic viruses are not subjected to a selection pressure dictated by nuclear splicing, allowing the presence of splicing-related sequences in viral genomes (Kamel et al. 2024). However, other viruses such as CHKV escape repression, and further investigation should focus on determining whether this resistance is due to mechanisms to antagonize U2 snRNA binding or the translocation of U2 snRNP to the cytoplasm.

Moreover, it is still unknown how U2 snRNP represses infection. The proteomic analysis of nucleus and cytoplasm showed that only U2 snRNP shows a robust relocation to the cytoplasm when compared to the other snRNPs (Kamel et al. 2024). Structural analyses revealed that the U2 snRNP interact with the target RNA sequence by a mixture of base-paring involving the U2 snRNA and protein binding. It is thus expected that the interaction between the U2 snRNP and the viral RNA will be strong and long-lived. Interestingly, removal of the branch point A causes U2 snRNP to stall on the intron (Zhang et al. 2021). Perfect branching point sequences lacking the unpaired A are also common in viral genomes (Kamel et al. 2024). Altogether, these findings suggest that U2 snRNP binding to viral RNA might end with a large protein–RNA complex being stalled on the viral RNA causing a rock block for processes such as viral replication or translation. However, further experiments are required to uncover the U2 snRNP antiviral mechanism.

## OUTLOOK

Increasing evidence suggests that nuclear RBPs are relocated to the cytoplasm upon infection with cytoplasmic RNA viruses. How this is achieved remains largely unknown for most viruses, except for picornaviruses and VSV, for which it was traced back to viral proteins targeting the NPC. With other viruses with no reported effects on the NPC such as SINV, the impact of infection in nucleo/cytoplasmic trafficking appears to be more subtle or selective. What causes this selective relocation of nuclear RBPs to the cytoplasm remains unknown, calling for further investigation.

The importance of relocated RBPs is evidenced by recent studies showing that they moonlight as antiviral factors (Sanz et al. 2015; Tapescu et al. 2023; Kamel et al. 2024). These findings point to a layer of intrinsic antiviral defences likely activated by virus-associated cellular stresses. Whether these nuclear RBPs cooperate or act independently requires further investigation. DDX39A (Tapescu et al. 2023) and U2 snRNP (Kamel et al. 2024) represent two examples of many nuclear RBPs that translocate to the cytoplasm in infected cells. Indeed, other proteins such as MATR3, NOP2, NOP53, and DDX21, showed antiviral function in preliminary functional assays (Kamel et al. 2024). These results highlight that the DDX39A and U2 snRNP are just the tip of the iceberg and other potential antiviral mechanisms remain to be discovered. For example, the nucleolar RBP NOP53 has been related to stress sensing (Lee et al. 2012), and its presence in SINV VROs may involve an unknown mechanism to detect virus infection. Moreover, it remains unknown to what extent viruses from different families are affected by these nuclear RBPs and whether the antiviral mechanisms of these proteins are conserved across species. Similarly, CHKV escapes the repression of some of these nuclear RBPs, but the mechanisms to antagonize them are unknown. By deepening our understanding of the molecular principles that underpin these emerging antiviral proteins, we should be able to unlock new avenues for research and develop new antiviral therapies.
